# Effect of Exercise Instructions With Ambulatory Accelerometer in Japanese Patients With Type 2 Diabetes: a Randomized Control Trial

**DOI:** 10.3389/fendo.2022.949762

**Published:** 2022-07-12

**Authors:** Jin Matsushita, Hiroshi Okada, Yuki Okada, Takashi Sekiyama, Hideto Iida, Atsushi Shindo, Hiroaki Murata, Michiaki Fukui

**Affiliations:** ^1^ Department of Rehabilitation, Matsushita Memorial Hospital, Moriguchi, Japan; ^2^ Department of Endocrinology and Metabolism, Graduate School of Medical Science, Kyoto Prefectural University of Medicine, Kyoto, Japan; ^3^ Department of Diabetes and Endocrinology, Matsushita Memorial Hospital, Moriguchi, Japan; ^4^ Department of Diabetes, Ikeda Hospital, Amagasaki, Japan; ^5^ Department of Orthopedic Surgery, Matsushita Memorial Hospital, Moriguchi, Japan

**Keywords:** ambulatory accelerometer, diabetes mellitus, exercise therapy, physical therapist, transtheoretical model

## Abstract

This study aimed to investigate the effects of physical therapists’ exercise instructions in Japanese patients with type 2 diabetes. Thirty-six participants were recruited from the outpatient clinic at Matsushita Memorial Hospital, Osaka, Japan from June 2020 to September 2020 and were randomly assigned to either the non-intervention or intervention group. The intervention group received exercise instructions from physical therapists for 30 min at baseline (week 0) and at week 4 by referring to ambulatory accelerometer records. Laboratory parameters, physical activity, body composition, motor skill, and transtheoretical model were assessed in both the groups at baseline (week 0) and week 8. In week 8, patients in the intervention group had a statistically significant reduction in HbA1c levels compared with those in the non-intervention group (7.3% [6.8-%–7.9%] vs. 7.4% [7.3%–7.7%], P = 0.04). The number of steps per day (P = 0.001), energy expenditure (P = 0.01), lower extremity muscle strength (P = 0.002), and 6-min walk test results (P = 0.04) were significantly increased in the intervention group compared with those in the non-intervention group in week 8. The transtheoretical model varied between baseline (week 0) and week 8 only in the intervention group (P < 0.001). Thus, outpatient exercise instructions from physical therapists could improve glycemic control owing to physical activity by improving motor skills and changing the transtheoretical model in Japanese patients with type 2 diabetes.

## Introduction

Prevention of the onset and progression of diabetic complications is a major issue worldwide from the perspectives of extending healthy life expectancy and reducing medical expenditure. The pathophysiology of type 2 diabetes mainly involves a decrease in insulin secretion capacity and an increase in insulin resistance. In diabetes treatment, improvement in insulin resistance by dietary therapy alone is limited, and it is important to combine it with exercise therapy (1). The combination of dietary and exercise therapy reduces the risk of diabetes by 30%–40% (2–4), and exercise therapy has attracted more attention in recent years since frailty and sarcopenia in older adults are becoming increasingly problematic. However, the rate of outpatient exercise therapy by physical therapists among patients with diabetes in Japan is low, and this issue needs to be addressed.

The low implementation rate of outpatient exercise therapy may be because of uncertainty about the effectiveness of exercise instructions from physical therapists. Previous studies showed the effect of supervised aerobic exercise in patients with type 2 diabetes in western countries (5). However, few studies have investigated the effects of supervised exercise in Japanese patients with type 2 diabetes. Moreover, the benefits of exercise might differ between the Japanese and western populations because of low body mass index (BMI) in Asian populations, which disrupts their insulin secretion capacity instead of building insulin resistance, a feature commonly observed in the Japanese population. Therefore, this randomized controlled study aimed to investigate the effects of exercise instructions by physical therapists in Japanese patients with type 2 diabetes.

## Materials and Methods

### Ethics

The study was approved by the local ethics committee of Matsushita Memorial Hospital (approval number: 19033) and conducted according to the principles of the Declaration of Helsinki. Written informed consent was obtained from all participants.

### Inclusion and Exclusion Criteria

Patients diagnosed with type 2 diabetes and who provided written consent were included in the study. Patients who did not provide written consent, those who were pregnant or breastfeeding, those whose HbA1c was 10% or higher, those receiving insulin therapy, those having contraindications to exercise therapy, and those who were deemed inappropriate by the investigator were excluded.

### Study Design

**Figure 1:**
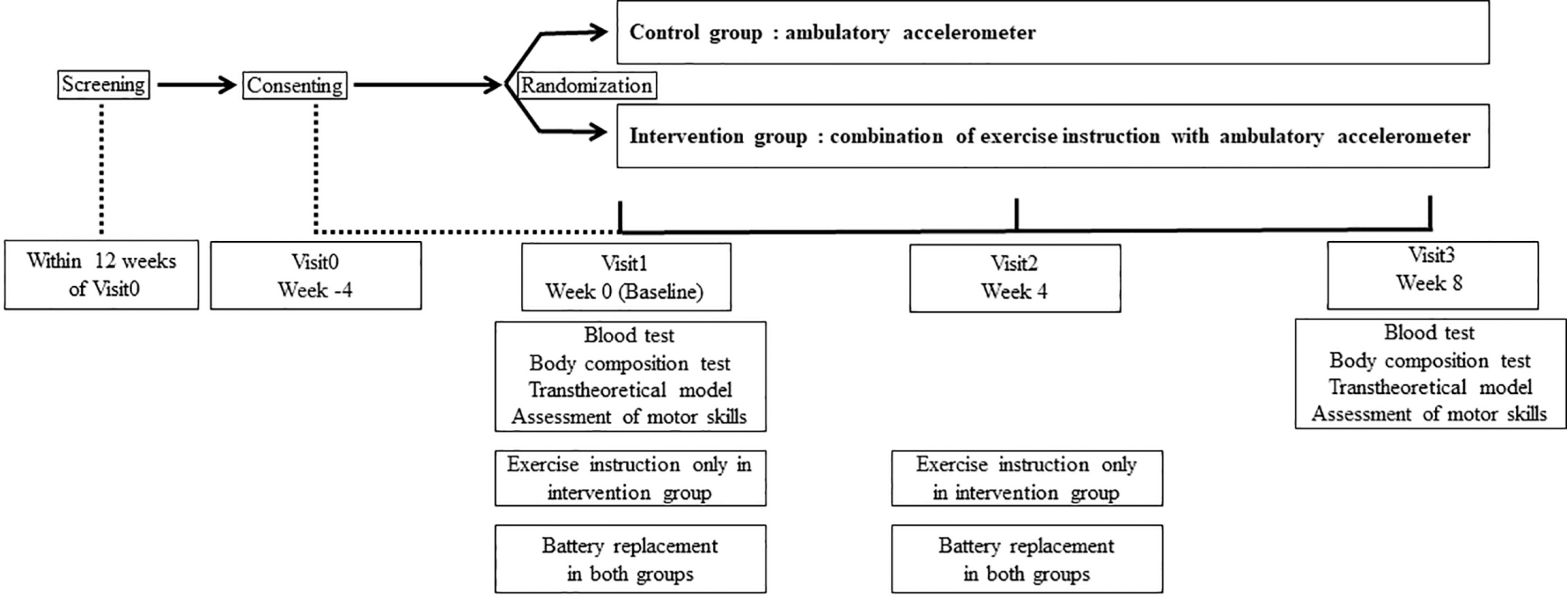
The study design.

Eligible trial participants were recruited from the outpatient clinic of Matsushita Memorial Hospital from June 2020 to September 2020 and were randomly assigned to either the non-intervention or intervention group by a third party using the envelope method. Primary care physicians were unaware of the group assignments. Laboratory parameters were measured at baseline (week 0) and visit 3 (week 8). The study design is illustrated in **Figure 1**. No medication changes were made during the study period.

### Sample Size

The null hypothesis of this study is defined as follows: the effect of the intervention group is the same as the effect of the non-intervention group. The alternative hypothesis of this study is defined as follows: the effect of the intervention group is greater than the effect of the non-intervention group. The minimum sample size required to achieve a significance of 0.05 for a one-sided t-test with a statistical power of 80% was used. Previous studies showed that HbA1c decreases by approximately 0.6% in the intervention group compared with that in the non-intervention group (6, 7). A sample size of 13 patients in one group was estimated to be sufficient. With an estimated dropout rate of 15%, the planned number of participants (36 participants, 18 in each group) was considered to have sufficient statistical value.

### Data Collection

Laboratory parameters were measured at baseline (week 0) and visit 3 (week 8). Serum total cholesterol and triglyceride concentrations were assessed using standard enzymatic methods. Hemoglobin A1c was assayed using high-performance liquid chromatography and expressed with the unit defined by the National Glycohemoglobin Standardization Program. Sitting blood pressure was measured after a 5-min rest at baseline (week 0) and visit 3 (week 8) in quiet space using an automatic device. Based on smoking habits, participants were classified as non-smokers and past or current smokers. A multifrequency impedance body composition analyzer (InBodyS10, Tokyo, Japan) was used to evaluate body composition at baseline (week 0) and visit 3 (week 8). InBodyS10 findings reportedly correlate well with dual-energy X-ray absorptiometry findings (8). Skeletal muscle mass index (kg/m^2^), dividing appendicular muscle mass (kg) by height squared (m^2^), was calculated. The transtheoretical model was devised and classified as precontemplation, contemplation, preparation, action, or maintenance and assessed by physical therapists at baseline (week 0) and visit 3 (week 8) (9).

### Assessment of Physical Activity and Motor skills

Physical activity, including the number of steps per day and energy expenditure, was evaluated at visit -1 (week -4) to baseline (week 0) and visit 2 (week 4) to visit 3 (week 8) using the Active style Pro HJA-750C (OMRON Corporation, Kyoto, Japan). Energy expenditure was defined as metabolic equivalent (MET)-hours per week (10). Active style Pro HJA-750C can measure energy expenditure per 10 sec using tri-accelerometer. Previous study reported the strong relationships between measured energy expenditure from tri-accelerometer and indirect calorimetry (11). The validity and accuracy of the Active style Pro HJA-750C and mechanism of measurement of energy expenditure have been described in detail elsewhere (11, 12). Motor skills, including handgrip strength, lower extremity muscle strength, and 6-min walk test scores, were evaluated at baseline (week 0) and visit 3 (week 8). Grip strength and lower extremity muscle strength were assessed as the average of maximum values of two times each side. Lower extremity muscle strength (6) was assessed by measuring the maximal strength of the quadriceps femoris muscles using a hand-held dynamometer (μTasF-1; ANIMA Corporation, Tokyo, Japan) whose interclass correlation coefficients (1,1) was 0.87 to 0.92 (7). The 6-min walk test scores were based on the distance walked by patients in 6 min.

### Intervention

The intervention group received exercise instructions from physical therapists for 30 min at baseline (week 0) and visit 2 (week 4) by referring to ambulatory accelerometer records. The physical therapist instructed the participants of the intervention group to walk for at least more than 150 min per week at moderate speed according to a guideline (13) and if possible, to walk more than 30 min per day. Both groups were provided ambulatory accelerometers (Active style Pro HJA-750C) during the observation period (week -4 to week 8) and were instructed to hold them when exercising or going out. The ambulatory accelerometer batteries were changed in both the groups at visit 2 (week 4).

### Primary and Secondary Endpoints

The primary endpoint was defined as the change in HbA1c levels during the intervention period. The secondary endpoint was defined as the changes in BMI, systolic blood pressure, diastolic blood pressure, fasting blood glucose, lipid profile, number of steps per day, energy expenditure, body composition, motor skill, and transtheoretical model.

The amount of change was defined as follows:

amount of change = post-intervention measurements (week 8) – pre-intervention measurements (week 0).

### Statistical Analyses

Participants enrolled in the study and assigned to the study treatment were defined as the largest analysis set (full analysis set [FAS]). The basic characteristics of the study participants were assessed for each group using FAS. To compare between the groups, Wilcoxon’s rank sum test was applied for continuous variables and Fisher’s exact test for categorical variables. Wilcoxon’s signed-rank test was performed for within-group comparison of changes. Spearman’s rank correlation coefficient test was performed to assess the association between the change in HbA1c and the change in number of steps per day, BMI, energy expenditure, appendicular muscle mass, body fat mass, skeletal muscle mass index, handgrip strength, lower-extremity muscle strength and 6-min walk test.

All continuous variables are presented as median ± interquartile range or absolute number. Differences were considered statistically significant at *P* values <0.05. Statistical analyses were performed using the JMP software, version 10 (SAS Institute, Cary, NC, USA).

## Results

**Figure 2:**
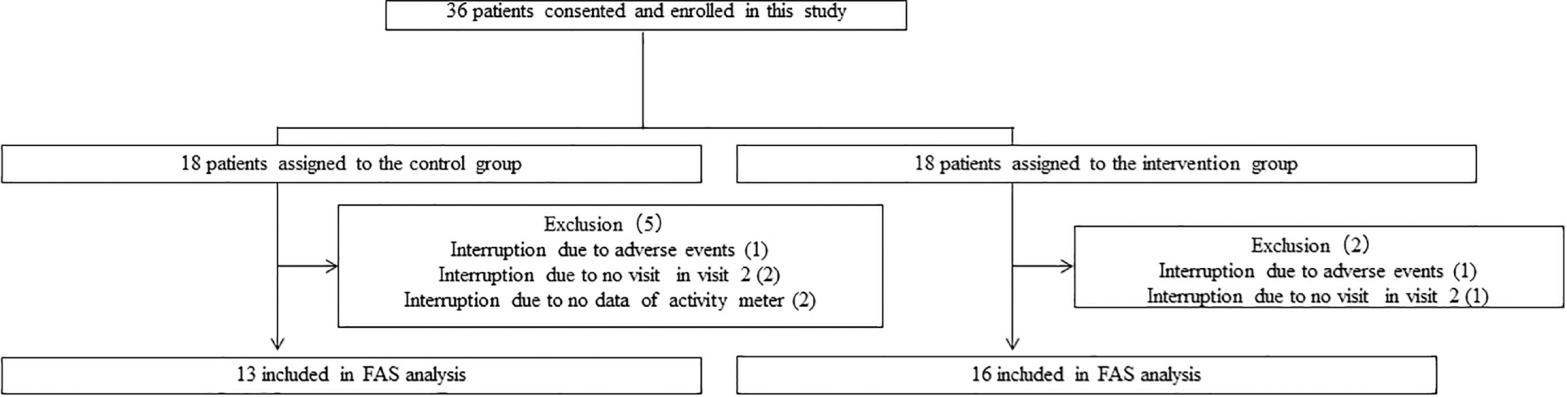
Study flow diagram for the registration of participants.

**Figure 2** shows the study flowchart. Thirty-six patients consented and were enrolled in this study. Participants who developed adverse events (n = 2; lumbago and cerebral infarction), did not visit a second time (visit 2) (n = 3), or had no ambulatory accelerometer data (n = 2) were excluded from the study. In the final analysis, 13 patients (non-intervention group) and 16 patients (intervention group) were included. The baseline characteristics of the study participants are presented in **Table 1**. The average HbA1c levels were 7.4% (7.3%–7.7%) and 7.3% (6.8%–7.9%) in the non-intervention and intervention groups, respectively, at baseline (week 0). We found no significant differences in any of the factors between the two groups at baseline (week 0).

**Table 1:** Characteristics at baseline (week 0).

	Non-intervention group	Intervention group	*p*
Age (year)	61 (50.5–68)	62 (51.5–77)	0.46
Sex (male/female)	11/2	14/2	0.82
Body mass index (kg/m^2^)	26.4 (22–31.1)	24.7 (22.5–28.7)	0.65
Duration of diabetes (year)	12 (2.5–18)	9 (2.3–14.8)	0.48
Systolic blood pressure (mmHg)	131 (125–1437)	129.5 (123–140.5)	0.88
Diastolic blood pressure (mmHg)	79 (72.5–82)	74 (69.3–85)	0.39
HbA_1_c (%)	7.4 (7.3–7.7)	7.3 (6.8–7.9)	0.15
Fasting plasma glucose (mg/dl)	149 (134–166)	141 (120–147)	0.14
Total cholesterol (mg/dl)	180 (156.5–192)	162 (144–182.5)	0.23
High density cholesterol (mg/dl)	47 (37–53)	40 (37–45.5)	0.22
Triglyceride (mg/dl)	120 (94.5–256)	136 (99.5–207.5)	0.61
Antidiabetic treatment (diet/oral hypoglycemic agent)	1/12	1/15	0.88
Smoking (never/past or current)	5/8	7/9	0.77
Number of steps per day (step)	3649 (2627–6313)	3951 (2700–4941)	0.98
Energy expenditure (MET h × week)	21 (15.4–28)	19.6 (14.5–31.7)	0.88
Appendicular muscle mass (kg)	22.4 (19.3–27.1)	21.2 (19.4–24.9)	0.46
Body fat mass (kg)	21.9 (13.9–34.7)	19.7 (17.6–29.3)	0.91
Skeletal muscle mass index (kg/m^2^)	7.8 (6.9–9.2)	7.5 (6.8–8.4)	0.31
Handgrip strength (kg)	37.9 (29.7–44.3)	32.1 (25.8–39.8)	0.39
Lower-extremity muscle strength (kgf/kg)	0.54 (0.42–0.66)	0.48 (0.36–0.53)	0.10
6-min walk test (m)	500 (454–544)	453 (405–510)	0.06
Transtheoretical model (precontemplation, contemplation, preparation, action or maintenance)	5/4/3/1	7/7/1/1	0.59

**Table 2** shows the changes in patient characteristics from baseline (week 0). BMI was similar between baseline (week 0) and week 8 in both the groups. A statistically significant treatment effect was found in glycemic control but not in blood pressure and lipid profile. Patients in the intervention group had a statistically significant reduction in HbA1c compared with that in those in the non-intervention group (P = 0.04). The number of steps per day (P = 0.001), energy expenditure (P = 0.01), lower extremity muscle strength (P = 0.002), and 6-min walk test results (P = 0.04) were significantly increased in the intervention group compared with those in the non-intervention group. The transtheoretical model was similar between baseline (week 0) and week 8 in the non-intervention group. However, the model varied between baseline (week 0) and week 8 in the intervention group (P < 0.001). The unadjusted regression analysis showed that the change in number of steps per day was significantly associated with the change in HbA1c (*r* = -0.55, P = 0.03). The change in BMI, energy expenditure, appendicular muscle mass, body fat mass, skeletal muscle mass index, handgrip strength, lower-extremity muscle strength and 6-min walk test were not associated with the change in HbA1c.

**Table 2:** Characteristics in week 8 and the change of characteristics from baseline.

	Week 8		Change from baseline		*p*
	Non-interventiongroup	Interventiongroup	Non-interventiongroup	Interventiongroup	
Body mass index (kg/m^2^)	26.5 (22.1–30.5)	24.8 (22.6–28.5)	- 0.2 (-0.3–0.7)	-0.1 (-0.3–0.5)	0.97
Systolic blood pressure (mmHg)	126 (116.5–139)	128 (123.3–133.3)	-9 (-13–5.5)	-2 (-8.8–2.8)	0.74
Diastolic blood pressure (mmHg)	84 (72–91.5)	76.5 (66.3–79)	3 (0–7.5)	-2.5 (-12.5–5.8)	0.10
HbA_1_c (%)	7.4 (7.1–8)	6.9 (6.6–7.3)	0 (-0.2–0.2)	-0.3 (-0.4–0)	0.04
Fasting plasma glucose (mg/dl)	151 (129.5–175)	136.5 (122.3–159.8)	39 (-74–60)	13.5 (-64.8–35.5)	0.23
Total cholesterol (mg/dl)	177 (156.5–190.5)	166.5 (156.8–185)	1 (-6–17.5)	3.5 (-6–14.8)	0.79
High density cholesterol (mg/dl)	44 (40.5–48.5)	43.5 (38.3–49.5)	0 (-3.5–3)	1.5 (0–2.8)	0.32
Triglyceride (mg/dl)	135 (92.5–222.5)	171 (84–203)	7 (-27.5–44.5)	-13 (-30.8–7.5)	0.35
Number of steps per day (step)	3246 (2579–6376)	8937 (6152–9814)	-559 (-1384–1743)	4054 (1742–6348)	0.001
Energy expenditure (MET h × week)	27.3 (15.4–41.3)	35.4 (29.1–50.8)	0.7 (-6.7–13.0)	17.5 (3.0–21)	0.01
Appendicular muscle mass (kg)	22.0 (19.4–26.9)	21.1 (18.6–23.6)	-0.2 (-0.7–0.6)	-0.3 (-0.8–0.1)	0.50
Body fat mass (kg)	22.4 (14.1–34.8)	20.2 (17.7–30.1)	0.1 (-0.8–1.2)	0.6 (-0.5–1.0)	0.71
Skeletal muscle mass index (kg/m^2^)	7.8 (7.0–9.2)	7.4 (6.6–8.1)	-0.1 (-0.3–0.2)	-0.1 (-0.3–0)	0.52
Handgrip strength (kg)	36.8 (27.8–42.3)	31.9 (26–38.4)	-0.6 (-1.8–0.9)	-0.4 (-1.6–0.8)	0.95
Lower-extremity muscle strength (kgf/kg)	0.54 (0.4–0.65)	0.55 (0.42–0.63)	0 (-0.05–0.02)	0.06 (0.01–0.09)	0.002
6-min walk test (m)	495 (467–506)	490 (443–525)	9 (-35–26)	25 (11–44)	0.04
Transtheoretical model (precontemplation, contemplation, preparation, action or maintenance)	5/5/2/1	0/2/0/14*	–	–	–

*P<0.0001, vs baleline.

## Discussion

In this study, we investigated the effect of exercise instructions from physical therapists using ambulatory accelerometers in Japanese patients with type 2 diabetes. Our major findings were as follows: glycemic control was better in the intervention group than in the non-intervention group, the intervention group had more improved motor skills than the non-intervention group, and the transtheoretical model varied in the intervention group but not in the non-intervention group between before and after intervention.

Walking is one of the most convenient and popular aerobic exercises for patients with type 2 diabetes. Recommendations for implementing exercise therapy include specific guidance on the type, duration, intensity, and frequency of exercise (14). A meta-analysis reported that at least 150 min of exercise per week is favorable for improved glycemic control (15). The Japanese diabetes treatment guideline also recommends aerobic exercise at moderate intensity for 150 min or more per week at least three times per week, with no more than 2 days of no exercise. In the current study, the number of steps per day and energy expenditure were sufficient to meet the guideline recommendations for the intervention group.

We showed that patients with supervised walking had significant reductions in HbA1c levels. However, this difference could not be entirely explained by participation in a supervised walking program alone. Behavioral changes and improved mobility owing to exercise instruction could also lead to increased and improved physical activity. In this study, the transtheoretical model varied between baseline (week 0) and week 8 only in the intervention group. Moreover, motor skills were greatly improved in the intervention group compared with those in the non-intervention group. When prescribing exercise therapy, instructions from a physical therapist with an ambulatory accelerometer as well as the loan of an ambulatory accelerometer might be effective. In other words, exercise therapy using only an ambulatory accelerometer may be less effective in self-care.

In this study, BMI was similar before and after the intervention, which is consistent findings of a previous study (16). A previous meta-analysis also reported that exercise could improve glycemic control, although no significant change in BMI was found for 18 weeks (16). There are some possible explanations for this finding. First, the intervention period of exercise could have been short, and the intensity of exercise could have been moderate. Second, the exercise intervention could have resulted in changes in food intake, with dietary intake possibly increasing in the intervention group. However, previous studies reported no significant differences in BMI between the exercise and diet group and the non-exercise and non-diet group (17, 18). Third, an increase in physical activity could have altered body composition. In other words, increased physical activity may reduce fat mass and increase muscle mass. In this study, fat mass and muscle mass were similar before and after the intervention, whereas motor performance differed significantly in the intervention group.

The finding that exercise can improve glycemic control without body weight reduction is important. Exercise can improve insulin resistance and chronic inflammation and reduce the accumulation of ectopic fat without body weight reduction (19–23). A previous report showed that a high-fat diet significantly increased intramyocellular lipids without body weight gain, resulting in worsening of insulin resistance in Japanese participants (24). In this regard, we suppose that increased and improved physical activity lead to improved insulin resistance owing to reduced ectopic fat accumulation, including intramyocellular lipids. Another explanation about improvement of glycemic control without body weight reduction is effect of exercise independent of insulin action. Insulin action causes glucose uptake *via* glucose transporter type 4. There is also a pathway for glucose uptake independent of exercise-induced insulin action. Intracellular adenosine monophosphate-activated protein kinase is activated by exercise, and this activation promotes glucose transporter type 4 translocation independent of insulin action (25).

This study had few limitations. The dietary intake may have affected our results. Unfortunately, there are no data on dietary intake. The number of 13 and 16 in each group was finally analyzed in this study. This study did not include intention to treat analysis because several people have dropped out. Our study population comprised Japanese people whose BMI was lower than that of the western population. Therefore, it is unclear whether our findings can be generalized to other ethnic groups.

In conclusion, outpatient exercise instructions from a physical therapist increased physical activity by improving motor performance and transtheoretical model. Outpatient exercise instructions by physical therapists with an ambulatory accelerometer can improve glycemic control in Japanese patients with type 2 diabetes.

## Data Availability Statement

The raw data supporting the conclusions of this article will be made available by the authors, without undue reservation.

## Ethics Statement

The studies involving human participants were reviewed and approved by the local ethics committee of Matsushita Memorial Hospital. The patients/participants provided their written informed consent to participate in this study.

## Author Contributions

All persons who fulfill the authorship criteria are listed as authors, and all authors certify that they have participated in this work sufficiently to take public responsibility for its content. JM and HO researched the data and wrote the manuscript. AS and HM contributed to the discussion. HO, JM, YO, TS, and HI researched the data and contributed to the conception and discussion. MF reviewed and edited the manuscript. All authors contributed to the article and approved the submitted version.

## Funding

This study was funded by Japanese Society of Hospital General Medicine.

## Conflict of Interest

HO received personal fees from MSD K.K., Mitsubishi Tanabe Pharma Corporation, Sumitomo Dainippon Pharma Co., Ltd., Novo Nordisk Pharma Ltd., Daiichi Sankyo Co., Ltd, Eli Lilly Japan K.K, Kyowa Hakko Kirin Company Ltd, Ltd, Kowa Pharmaceutical Co., Ltd, Ono Pharmaceutical Co., Ltd. and Sanofi K.K. MF received grants from Ono Pharma Co. Ltd., Oishi Kenko inc., Yamada Bee Farm, Astellas Pharma Inc., Mitsubishi Tanabe Pharma Corp., Nippon Boehringer Ingelheim Co. Ltd., MSD K.K., Kissei Pharma Co. Ltd., Daiichi Sankyo Co. Ltd., Sanwa Kagagu Kenkyusho Co., Ltd., Sanofi K.K., Kyowa Kirin Co., Ltd., Sumitomo Dainippon Pharma Co., Ltd., Terumo Corp., Tejin Pharma Ltd., Novo Nordisk Pharma Ltd., Eli Lilly, Japan, K.K., Taisho Pharma Co., Ltd., Abbott Japan Co. Ltd., Nippon Chemiphar Co., Ltd., Kowa Pharma Co. Ltd. and Johnson & Johnson K.K. Medical Co. and received personal fees from Abbott Japan Co. Ltd., Kissei Pharma Co., Ltd., Sumitomo Dainippon Pharma Co. Ltd., Mitsubishi Tanabe Pharma Corp., Daiichi Sankyo Co. Ltd., Sanofi K.K., Astellas Pharma Inc., MSD K.K., Kyowa Kirin Co. Ltd., Taisho Pharma Co., Ltd., Kowa Pharma Co. Ltd., Mochida Pharma Co. Ltd., Novo Nordisk Pharma Ltd., Ono Pharma Co. Ltd., Sanwa Kagaku Kenkyusho Co. Ltd., Eli Lilly Japan K.K., Bayer Yakuhin, Ltd., AstraZeneca K.K., Nippon Boehringer Ingelheim Co., Ltd., Teijin Pharma Ltd., Medtronic Japan Co. Ltd., Arkray Inc. and Nipro Corp. outside the submitted work.

The remaining authors declare that the research was conducted in the absence of any commercial or financial relationships that could be construed as a potential conflict of interest.

## Publisher’s Note

All claims expressed in this article are solely those of the authors and do not necessarily represent those of their affiliated organizations, or those of the publisher, the editors and the reviewers. Any product that may be evaluated in this article, or claim that may be made by its manufacturer, is not guaranteed or endorsed by the publisher.
